# Autophagy in synapse formation and brain wiring

**DOI:** 10.1080/15548627.2023.2179778

**Published:** 2023-03-02

**Authors:** Bassem A. Hassan, P. Robin Hiesinger

**Affiliations:** a Institut du Cerveau-Paris Brain Institute (ICM), Sorbonne University, Inserm, CNRS, Hôpital Pitié Salpêtrière, Paris, France; b Division of Neurobiology, Free University of Berlin, Berlin, Germany

**Keywords:** Active zone, brain development, filopodia, neuron, neuronal autophagy, synapse, synaptic autophagy, synaptogenesis

## Abstract

A recent characterization of the role of autophagy in two different neuron types during brain development in *Drosophila* revealed two different mechanisms to regulate synapse formation. In photoreceptor neurons, autophagosome formation in synaptogenic filopodia destabilizes presumptive synaptic contacts and thereby restricts incorrect synaptic partnerships. In dorsal cluster neurons, autophagy is actively suppressed to keep mature synapses stable during axonal branching. These findings indicate that different neuron types can require activation or suppression of synaptic autophagy during the same developmental period to ensure proper synapse formation and brain connectivity.

The role of macroautophagy/autophagy in the brain was initially characterized in mice with neuron-specific loss of the core autophagy genes *Atg5* or *Atg7*. This seminal work revealed that functional neurons develop in the absence of autophagy, but are at increased risk for adult-onset degeneration. However, subsequent studies revealed that autophagy-deficient neurons, although functional, exhibit hallmarks of neurodevelopmental defects. Loss of autophagy during mouse brain development leads to increased spine density due to defective pruning after synapse formation, a hallmark of neurodevelopmental autism spectrum disorders. Conversely, in worms and flies, loss of neuronal autophagy can lead to reduced synapse formation. These findings suggest developmental roles for autophagy at the level of synapses, yet there is currently no coherent picture about a single, shared mechanism by which autophagy functions during brain development and synapse formation.

Our labs have recently studied the role of neuronal autophagy during fly brain development in two different neuronal cell types. Loss of autophagy in photoreceptor neurons leads to a stabilization of synaptogenic filopodia, which in turn leads to increased synapse formation, similar to what has been suggested for spine stabilization in mice (Figure 1A). Importantly, for fly photoreceptor neurons it could be shown that the increased number of synapses includes synaptic partnerships not present in wild-type brains, suggesting a role for autophagic regulation as part of the genetic program for synapse-specific brain wiring. In these axon terminals, autophagy specifically occurs at the tips of filopodia, leading to filopodial destabilization and thereby increased filopodial dynamics that restrict synaptic partner choice (Figure 1A). In a recent study, we have now expanded the analysis of a loss of neuronal autophagy to a second, very different neuron type: dorsal cluster neurons (DCNs) [1]. These large interneurons project contralaterally through the entire central brain to compare the visual information between the left and right hemispheres. In contrast to the unbranched photoreceptor axons, DCN axons undergo a complicated morphogenetic program that leads to stereotypic axonal branching patterns (Figure 1B). If autophagy can destabilize axon filopodial projections during development, how does a loss of autophagy affect the development of such a morphologically complicated axonal structure?

**Figure 1.**
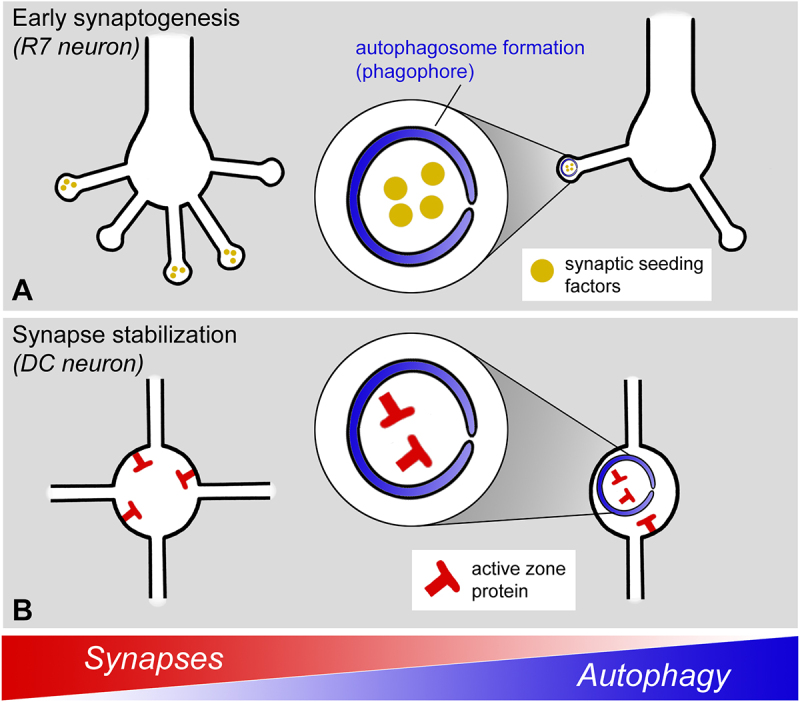
Regulation of synapse formation through two different mechanisms in two neuron types in Drosophila. (A) In fly photoreceptor neurons, autophagosome formation is selectively triggered at the tips of synaptogenic filopodia to regulate synapse numbers and partnerships. (B) In fly dorsal cluster neurons, autophagy is actively suppressed to stabilize synapses and branch formation.

Surprisingly, loss of autophagy has no obvious effect on the morphology or synapse numbers of DCN axonal projections; instead, normal axonal branching patterns and synapses only develop under conditions of active *suppression* of autophagy. The signaling mechanism underlying this suppression requires Egfr (Epidermal growth factor receptor) function, which had previously been shown to function in DCN axon branching during early axon development. The new study now revealed an independent second, later role for Egfr signaling in axonal branches during synapse formation. However, loss of Egfr signaling does not affect the initial trafficking of presynaptic proteins to synaptogenic filopodia at the time synaptogenesis commences in these branches. Instead, Egfr function is specifically required to stabilize newly formed presynaptic active zones by preventing their turnover through membrane degradation (Figure 1B).

Mature presynaptic sites are marked by the active zone scaffold protein Brp. Loss of Egfr function leads to increased colocalization of Brp with markers for endomembrane degradation, including the early autophagosomal marker Atg5, the late endosomal marker Rab7, and the lysosomal marker spin (spinster). Correspondingly, a reduction of endomembrane degradation through *Rab7* knockdown as well as a reduction of autophagy through *Atg6* knockdown partially rescue the synaptic connectivity defects associated with loss of Egfr function. These findings suggest autophagic degradation and turnover of active zone proteins as a default state that is actively suppressed during a critical developmental period of brain wiring in DCNs.

Presynaptic active zones form concomitantly with the elaborate axonal branch morphogenesis of DCN axons. A role for synapse formation in axonal and dendritic branching is well known as “synaptotropic growth”. During synaptotropic growth synapse formation stabilizes branches and provides a local basis for new probabilistic branch formation. Correspondingly, suppression of autophagy during DCN synapse formation secondarily leads to branch destabilization and a deterioration of the stereotypic axonal branching pattern (Figure 1B). The consequences of increased synaptic autophagy during this critical period of DCN development thereby include altered axonal morphology, synaptic connectivity and visual behavior.

We conclude that autophagy can restrict synapse formation through different mechanisms in two different neuron types of the fly visual system during the development of synaptic connectivity. In the non-branched axon terminals of photoreceptor neurons autophagy is actively induced at the tips of synaptogenic filopodia to restrict synapse formation and partner choice; in the highly branched axons of DCNs autophagy is actively suppressed to allow synaptotropic growth of an axonal branching pattern. Furthermore, the autophagic cargo in photoreceptor filopodia include the presynaptic seeding factors Liprin-a and RhoGAP100F/Syd-1, but not the mature active zone marker Brp (Figure 1A); by contrast, autophagic degradation induced by loss of Egfr function targets mature, brp-containing active zones (Figure 1B). These findings support a view of autophagy as a regulatory module during brain development that can be employed in a cell type-specific manner

The observation that activation versus suppression of autophagy can be required in two different neuron types in the same brain region and during the same critical window of brain development may also affect how we view brain-wide autophagic up- or downregulation as a therapeutic opportunity. If neighboring neurons employ opposite autophagic regulation to develop proper synaptic connectivity, then levels of autophagy must be controlled at the level of individual neurons to ensure normal development.

In summary, we have identified two very different neuron types in the fly visual system that employ the spatial and temporal regulation of autophagy for the fine-tuning of synaptic connectivity through selective restriction of synapse formation, albeit through different mechanisms. In both cases cell type-specific modulation of presynaptic autophagy during synapse formation represents a quantitative contribution to brain wiring that is selectable at the level of animal behavior. Based on the diversity of autophagy-dependent synapse development in the few neuron types analyzed thus far, we speculate that more cell-specific roles of autophagy will emerge as more neuron types are investigated during brain development.
